# Amsterdam urban canals contain novel niches for methane‐cycling microorganisms

**DOI:** 10.1111/1462-2920.15864

**Published:** 2021-12-13

**Authors:** Koen A. J. Pelsma, Michiel H. in 't Zandt, Huub J. M. Op den Camp, Mike S. M. Jetten, Joshua F. Dean, Cornelia U. Welte

**Affiliations:** ^1^ Department of Microbiology Radboud Institute for Biological and Environmental Sciences, Heyendaalseweg 135 Nijmegen 6525 AJ The Netherlands; ^2^ Netherlands Earth System Science Centre Utrecht University, Heidelberglaan 2 Utrecht 3584 CS The Netherlands; ^3^ Soehngen Institute of Anaerobic Microbiology Radboud University, Heyendaalseweg 135 Nijmegen 6525 AJ The Netherlands; ^4^ School of Environmental Sciences University of Liverpool Liverpool L69 3GP UK

## Abstract

Urbanised environments have been identified as hotspots of anthropogenic methane emissions. Especially urban aquatic ecosystems are increasingly recognised as important sources of methane. However, the microbiology behind these emissions remains unexplored. Here, we applied microcosm incubations and molecular analyses to investigate the methane‐cycling community of the Amsterdam canal system in the Netherlands. The sediment methanogenic communities were dominated by *Methanoregulaceae* and *Methanosaetaceae*, with co‐occurring methanotrophic *Methanoperedenaceae* and *Methylomirabilaceae* indicating the potential for anaerobic methane oxidation. Methane was readily produced after substrate amendment, suggesting an active but substrate‐limited methanogenic community. Bacterial 16S rRNA gene amplicon sequencing of the sediment revealed a high relative abundance of Thermodesulfovibrionia. Canal wall biofilms showed the highest initial methanotrophic potential under oxic conditions compared to the sediment. During prolonged incubations the maximum methanotrophic rate increased to 8.08 mmol g_DW_^−1^ d^−1^ that was concomitant with an enrichment of *Methylomonadaceae* bacteria. Metagenomic analysis of the canal wall biofilm lead to the recovery of a single methanotroph metagenome‐assembled genome. Taxonomic analysis showed that this methanotroph belongs to the genus *Methyloglobulus*. Our results underline the importance of previously unidentified and specialised environmental niches at the nexus of the natural and human‐impacted carbon cycle.

## Introduction

Since the Industrial Revolution, atmospheric greenhouse gas (GHG) concentrations have been steadily increasing due to human activities like cattle farming, intensive agriculture, use of synthetic fertilisers, waste management and fossil fuel burning (Schaefer *et al*., 2016; Saunois *et al*., 2020). Even though the current atmospheric methane (CH_4_) concentration of >1.87 ppm is lower than the >416 ppm carbon dioxide (CO_2_) concentration (Dlugokencky, 2020), CH_4_ accounts for the equivalent of 60% of the radiative forcing of CO_2_ due to its 86 times higher global warming potential over a 20‐year time‐scale (Myhre *et al*., 2013; Dean *et al*., 2018; Nisbet *et al*., 2019). A total of 306 Tg yr^−1^ of CH_4_ is emitted by freshwater ecosystems such as lakes, ponds and wetlands globally (Kirschke *et al*., 2013; Saunois *et al*., 2020). Wetlands comprise 40% of natural CH_4_ emissions, whereas other freshwater systems are now thought to be as high as 159 Tg yr^−1^ or 43% of global natural CH_4_ emissions (Bastviken *et al*., 2011; Saunois *et al*., 2020).

An important understudied aspect of freshwater CH_4_ emissions is the influence of urbanisation on the GHG emissions of the surrounding aquatic systems. Many freshwater sources have been attractive locations for human settlements, which led to the majority of cities containing waterways. The United Nations report that currently an estimated 54% of the human population is living in cities and this percentage is estimated to grow to 66% by 2050 (United Nations, 2015). Microorganisms tend to be more abundant in urban waters due to the combined sewer overflows or discharge from wastewater treatment plants (Young and Thackston, 1999; Hladilek *et al*., 2016; Price *et al*., 2018; Mansfeldt *et al*., 2020). In addition, leaking natural gas and sewer pipes, as well as stormwater, influences available substrates for microbial communities in cities (Lamb *et al*., 2016; Smith *et al*., 2017; McLellan and Roguet, 2019). All these changes are consistent across waterways, so the term ‘urban stream syndrome’ was coined to describe these changes (Meyer *et al*., 2005). A recent analysis of published CH_4_ emission data from streams and rivers revealed that CH_4_ concentrations within urban waters rival those of wetlands and agricultural streams (Stanley *et al*., 2016). Furthermore, an analysis of diffusive CH_4_ fluxes from various ecosystems revealed that, like wetlands, urban waterways have higher CH_4_ emissions than non‐urbanised rivers and streams. Changes in nutrient loading caused by human activity, together with increased CH_4_ concentrations, suggest that urbanisation leads to an imbalance between CH_4_ production and consumption resulting in net emissions of CH_4_.

CH_4_ concentrations and emissions from freshwaters have been reported for several riverine systems in Europe (Alshboul *et al*., 2016; Borges *et al*., 2018; Marescaux *et al*., 2018; Brown and Hershey, 2019; Herrero Ortega *et al*., 2019), China (Wang *et al*., 2018; Wang *et al*., 2021) and the United States (Brigham *et al*., 2019). The majority of studies find a positive correlation with temperature and dissolved CH_4_ during summer. However, other environmental parameters, like degree of eutrophication, are not always correlated to increased CH_4_ concentrations or emissions (Herrero Ortega *et al*., 2019). Several studies posit that the increased concentrations within cities are due to wastewater treatment plant effluent and not due to production in the river sediment (Alshboul *et al*., 2016; Wang *et al*., 2018). A recent study of built canals in urban and agricultural environments showed CH_4_ emissions for these systems as high as tropical wetlands, more than freshwater lakes (Peacock *et al*., 2021). Thus, urban environments can be considered understudied hotspots of microbial CH_4_ cycling.

Most of the CH_4_ from riverine and urban aquatic ecosystems is thought to be biogenic (Schaefer *et al*., 2016; Zazzeri *et al*., 2017). Biological CH_4_ production is considered the last step in the anaerobic fermentative degradation of organic matter and is performed by methanogenic archaea (Conrad, 2009). Not all CH_4_ produced in anaerobic environments enters the atmosphere. A majority is converted to CO_2_ by aerobic and anaerobic methanotrophs, diminishing the climate impact (Knittel and Boetius, 2009; Knief, 2015). Therefore, insight into the microbial CH_4_ cycle is paramount to understanding balances in CH_4_ emissions. Until now urban microbiome research has mainly focused on planktonic cells in the water column (Savio *et al*., 2015; Medeiros *et al*., 2016; Cannon *et al*., 2017; Hosen *et al*., 2017; Fresia *et al*., 2019), whereas methanogens reside in the anoxic sediments of urban waters. However, the studies outlined above reported differences in microbial community structure in urban waters compared to rural waters. Studies that also took samples of sediments observe a similar trend, with sediment microbial communities changing in response to increased nutrient input associated with urbanisation (Saxena *et al*., 2015; Hosen *et al*., 2017; Saxena *et al*., 2018). So far, no investigation into the community structure of CH_4_‐cycling microorganisms in urban waterways has been undertaken.

Here, we describe the urban microbial community of the Amsterdam canals, in the Netherlands, to investigate the local CH_4_ cycle of these heavily urbanised waterways. We provide a general description of the microbial community accompanied by microcosm‐based rate measurements of the methane‐cycling bacteria and archaea. Our study reveals that canal wall biofilms, a niche for aerobic *Methyloglobulus* methanotrophs, might form an as yet underestimated CH_4_ filter in urbanised environments.

## Results

### Biogeochemistry of sample sites

Nitrate, ammonium, phosphate and total organic carbon (TOC) levels were similar for each sample site and indicated an oligotrophic water column (Table 1). A difference in salinity was measured at the Artis site. This location is closest to the IJ, which is the brackish canal directly North of Amsterdam's city centre. There is a daily influx of brackish water from the IJ when the sluice gates at IJmuiden are closed, an effect that is more pronounced in drier periods when the water level is low (*Yu et al*., 2018a). *In situ* dissolved oxygen concentrations showed a water column that was well‐mixed and oxygenated (5.5 ± 0.9 mg L^−1^) down to the water–sediment interface. The Bloemgracht sampling site was most depleted in O_2_ with a bottom water O_2_ concentration of 3.1 mg L^−1^. Apart from a higher electrical conductivity of 5057 μS cm^−1^ at the Artis site, the remaining sites were of similar water chemistries even though they were located across the central Amsterdam canal network (Table 1, Supplementary Table S1).

**Table 1:** Physicochemical analysis of the sampled canal surface waters.

Site	Coordinates	Canal depth (m)	DO (mg L^−1^)	Electrical conductivity (μS cm^−1^)	pH	Temperature (°C)	NO_3_^−^ (μM)	NH_4_^+^ (μM)	PO_4_^3−^ (μM)	TOC (mg L^−1^)	CH_4_ (μmol L^−1^)
Bloemgracht	N 52.374064 E 4.878169	1.96	3.1	1979	7.7	22	52	10	2	13	0.04–0.15
Amstel	N 52.356174 E 4.905305	1.13	6.6	1204	8.0	24	64	8	2	13	0.13–0.52
Artis	N 52.366912 E 4.91839	1.84	5.2	5057	7.8	23	62	6	1	10	0.11–0.54
Prinsengracht	N 52.372003 E 4.882714	1.54	4.6	1985	7.9	23	57	8	2	15	0.3–0.42
Amstelsluizen	N 52.362367 E 4.902534	1.99	5.5	2384	7.9	23	62	5	2	11	0.4–0.59

Depth, dissolved oxygen, salinity, pH and temperature were measured *in situ*. Data presented are from one independent measurement per sample site. Dissolved CH_4_ is presented as the range of three independent measurements. DO, dissolved oxygen; NO_3_^−^, nitrate; NH_4_^+^, ammonium; PO_4_^3−^, phosphate; TOC, total organic carbon.

### Microcosm incubations highlight methanogenic potential of canal sediments

**Fig. 1:**
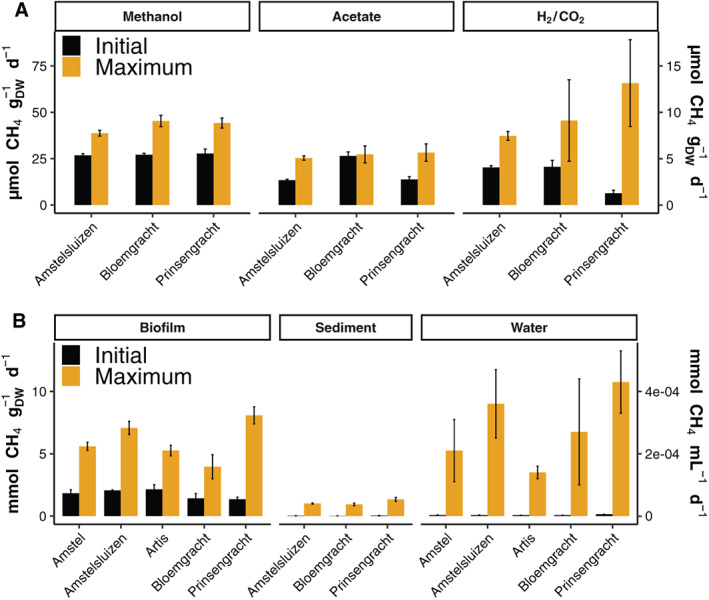
(A) Rates of methanogenesis measured in the first 5 days (initial) and the enriched rate after substrate amendment (maximum). Initial rates are plotted with respect to the secondary *y*‐axis on the right. The primary *y*‐axis displays the maximum methanogenic rate. B. CH_4_ oxidation calculated from the decrease or increase of CH_4_ over time for the first days of the microcosm incubations (initial) and the maximum measured rate. Each bar indicates the mean slope of at least two linear least‐squares regressions and the corresponding deviation from the mean. Biofilm and sediment rates are expressed in mmol g_DW_^−1^ d^−1^ and the water rate is expressed in mmol L^−1^ d^−1^. Methanogenic rates in amended cultures are expressed as μmol g_DW_^−1^ d^−1^.

Since dissolved CH_4_ in urban waters tended to be higher compared to rural areas, we hypothesised that urban waterways may be a novel niche for CH_4_‐cycling microorganisms (Wang *et al*., 2018; Brigham *et al*., 2019; Peacock *et al*., 2021). To determine if both methanogens and methanotrophs form an active part of the urban aquatic microbial community we incubated environmental samples in microcosms and followed the change of CH_4_ over time. Sediments from the Bloemgracht, Prinsengracht and Amstelsluizen sites were amended separately with canal water sampled from the respective sampling sites. Microcosm incubations to determine methanogenic activity were done only with sediment slurries as the water column was completely oxygenated (Table 1). Methanogenic potential was determined for three canonical substrates with H_2_/CO_2_, acetate, or methanol and a control without substrate (Fig. S2). Production of CH_4_ was measured in the first week of incubation for all substrates. After 10 days, the amount of headspace CH_4_ in microcosms amended with methanol and acetate remained constant, indicating complete substrate consumption. Headspace CH_4_ in microcosms supplied with H_2_/CO_2_ increased steadily with time. Upon addition of more substrate, all incubations showed a sharp increase in produced CH_4_. This observation could be repeated two times after adding new substrate. Remarkably, unamended sediment did not produce CH_4_ at detectable levels, indicating labile organic matter fractions were depleted or were consumed during transportation and storage. The highest initial metabolic potentials were determined for sediments incubated with methanol, approximately 5.5 μmol CH_4_ g_DW_^−1^ d^−1^ (Fig. 1A & S2). For microcosms amended with acetate, Bloemgracht sediment showed a two times higher initial potential rate (5.3 μmol CH_4_ g_DW_^−1^ d^−1^) compared to Prinsengracht and Amstelsluizen sediment.

### Methanotrophy can be readily activated in canal wall biofilm microcosms

To determine the methanotrophic metabolic potential of sampled canal waters and biofilms, microcosm incubations of canal water and biofilm were performed with a headspace containing 0.9 mmol L^−1^ CH_4_. Within 3 days, all five biofilm microcosms showed rapid CH_4_ conversion (Fig. S3A). The amount of O_2_ in the bottles was not sufficient to completely consume all the added CH_4_. Therefore, the bottles were flushed with filter‐sterilised air and the headspace concentration of CH_4_ was adjusted to 0.38 mmol L^−1^ CH_4_ for the remainder of the incubation period. After addition of fresh CH_4_, it was readily consumed and the headspace was replaced two more times.

Water column methanotrophy was measured over 80 days and showed large variability between the triplicate bottles (Fig. S3A). During the first 30 days of the microcosm incubation a steady decrease of CH_4_ could be observed. After 40 days, the Bloemgracht, Prinsengracht and Amstelsluizen microcosms consumed CH_4_ at an increased rate in two of the three replicate microcosms. This indicated growth of methanotrophs in the water and confirmed their presence in the water column (Supplementary Fig. S4).

Comparing initial methanotrophic rates between the biofilm, sediment and water incubations showed a distinctly higher rate for the biofilms (Fig. 1A). Normalised to g_DW_ the metabolic potential for methanotrophy in all five biofilms was in a range of 1.35–2.14 mmol g_DW_^−1^ d^−1^. Initial rates for the sediment methanotrophs were around 0.03 mmol g_DW_^−1^ d^−1^ indicating that this metabolic potential is present in the sediment as well as the biofilm. Nevertheless, this sediment methanotrophic rate is high enough to oxidise the CH_4_ produced in the sediment if ample oxygen is available and ebullitive (bubble) CH_4_ flux is low. Moreover, the water content of the biofilm was higher than that of the sediment thus influencing the normalisation. The methanotrophic rate of the water column ranged between 0.003 and 0.006 mmol L^−1^ d^−1^.

Since there was detectable oxygen at the water–sediment interface in each canal (Table 1), sediments were incubated under oxic conditions with an 8.5% CH_4_ headspace to determine the aerobic methanotrophic potential. The oxic microcosms consumed between 15% and 20% of the added CH_4_ within the first week, and after 16 days the methanotrophic rate increased sharply. Upon refreshing the headspace with filter‐sterilised air and CH_4_, the microcosms consumed all CH_4_ within 7 days at a maximum measured rate of 1.34 mmol g_DW_^−1^ d^−1^.

### The microbial community in Amsterdam canals shows great metabolic flexibility

**Fig. 2:**
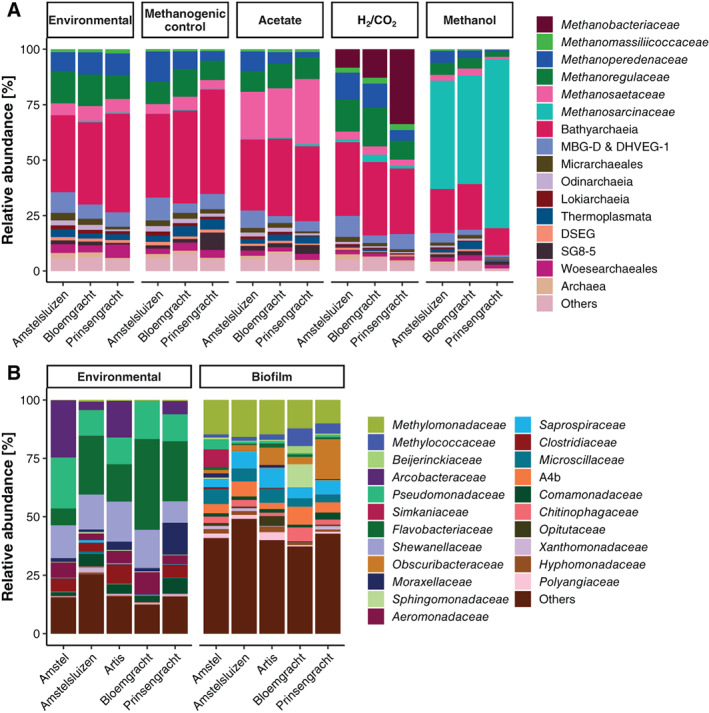
Archaeal community compositions of the sediment (A) and bacterial compositions of the biofilm (B) based on 16S rRNA gene amplicon sequencing. Environmental (initial) compositions are presented next to the amended incubations. Whenever possible taxonomy is represented at the family level. ASVs that averaged fewer than 1% of all reads were grouped into the category ‘Others’.

The incubation experiments highlighted the metabolic potential of the CH_4_‐cycling community within the urban canal system of Amsterdam. Using 16S rRNA gene amplicon sequencing we profiled the archaeal and bacterial communities of the environmental samples and the final state of the microcosm incubations after significant substrate was converted. For the methanogenic community analysis, we focused on the archaeal community (Fig. 2A). A high degree of similarity was found for the environmental archaeal community of all three sediment sample sites. Methanogens belonging to the families *Methanoregulaceae* and *Methanosaetaceae* were the most abundant. This suggests that hydrogenotrophic and acetoclastic methanogenesis could be accounting for the majority of the produced CH_4_ in the canal sediments. Approximately 10% of the total archaeal community was classified as ‘*Candidatus* Methanoperedens’, an anaerobic NO_3_^−^‐dependent CH_4_ oxidiser. A large part of the archaeal community was assigned to the metabolically versatile Bathyarchaeia. The archaeal community changed dramatically over the course of the methanogenic incubations. Specifically, the bottles amended with methanol enriched considerably for *Methanosarcinaceae*. Surprisingly, H_2_/CO_2_ amendment led to growth of hydrogenotrophic *Methanobacteriaceae* instead of the initially present *Methanoregulaceae*. During the incubations, other archaeal and bacterial community members did not seem to change much, and consisted of *Anaerolineaceae* and Thermodesulfovibrionia (Fig. 2A and Supplementary Fig. S5, Supplementary Table S3). 16S rRNA gene qPCR analysis of the environmental DNA yielded a ratio of archaea to bacteria of ~1:12 for the sediment samples, of ~1:3–25 for the water samples and of ~1:70–200 for the biofilm samples (Supplementary Fig. S8). No archaeal amplicons could be obtained for the biofilm and water samples because the constructed sequencing libraries did not pass quality control repeatedly, possibly due to a low amount of archaeal DNA. The qPCR results showed archaea to be present in low abundance, and metagenomic analysis of the biofilm (which was not performed for the water samples) using phyloFlash revealed that only 0.035% of the recovered 16S rRNA gene sequences were of archaeal origin.

**Fig. 3:**
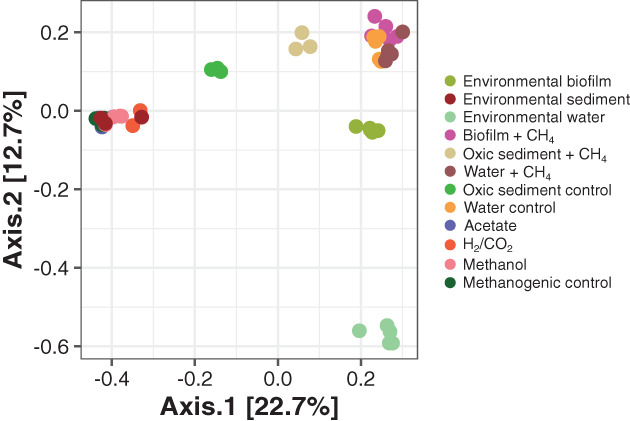
Principal coordinate analysis of all bacterial ASVs for all samples and incubations. Ordination was performed based on Bray–Curtis dissimilarity in R (v3.6.3; R Core Team, 2019) with the package *phyloseq* (McMurdie and Holmes, 2013). Colours represent the different amendments.

The bacterial community of the biofilm changed from one dominated by *Flavobacteriaceae*, *Shewanellaceae* and *Pseudomonaceae* to a community where 30% was *Methylococcaceae* and *Methylomonadaceae* (Fig. 2B). Interestingly, in the biofilm of the Amstel location around 15% of the total community was classified as *Simkaniaceae* of the Chlamydiae phylum. While the aerobic methanotrophs were approximately 0.7% of the initial bacterial biofilm community, their rapid consumption of CH_4_ in the microcosm incubations indicated a potential for a rapid activation of CH_4_ metabolism. A similar result was obtained for the bacterial community after the oxic incubation of the sediment. After 53 days of incubation a strong enrichment of *Methylococcus* was seen, with a small enrichment of ‘*Ca*. Methylospira’ (Fig. S6). To ascertain the changes in the bacterial community a principal coordinate analysis based on Bray–Curtis dissimilarity was performed (Fig. 3). A clear clustering took place based on whether O_2_ was present as the sediment samples grouped together and moved toward a community more like the incubated biofilm. This pattern was expected as incubating with CH_4_ and O_2_ are conditions selecting for aerobic methanotrophs. The high degree of similarity of the bacterial community in the amended methanogenic cultures suggested that the archaeal community dominated activity under these conditions.

### Metagenome sequencing reveals a novel methanotroph

**Fig. 4:**
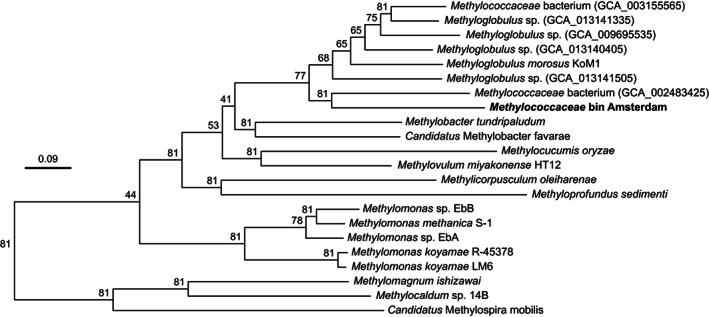
Phylogenomic placement with respect to representatives of the *Methylococcaceae* family of the obtained *Methylococcaceae* bin (in bold) computed using UBCG2 (Kim *et al*., 2021). Reference genomes were obtained from the NCBI Assembly database on February 12, 2021. The tree was generated with RAxML (Stamatakis, 2014) and the node values indicate the gene support index as calculated by UBCG2.

**Fig. 5:**
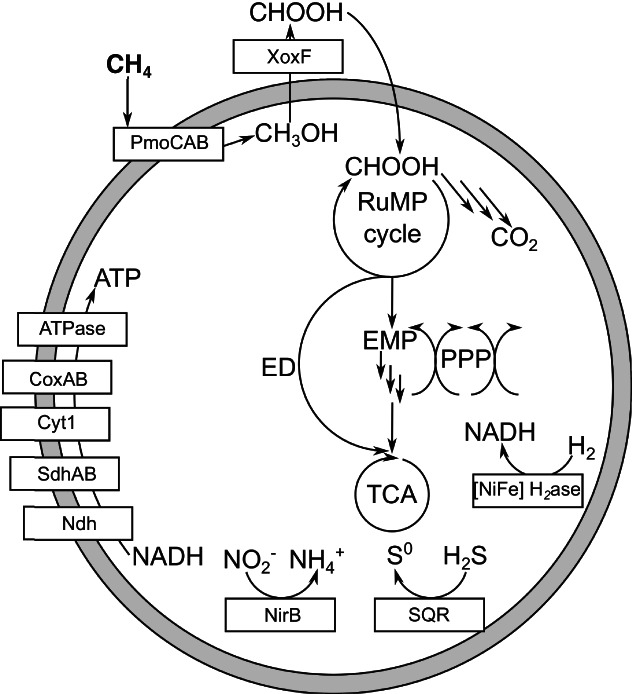
Schematic representation of the genomic metabolic potential found in the *Methyloglobulus* metagenome‐assembled genome of the canal wall biofilm.

In addition to the 16S rRNA gene amplicons, whole metagenomes of the biofilms and the sediments were sequenced. After automated binning, we recovered a metagenome‐assembled genome (MAG) of a novel *Methylococcaceae* methanotroph with 84% completeness and 6.6% contamination (Table S2). This was the only methanotrophic bin we obtained and an HMMer search for *pmoA* did not lead to the identification of additional methanotrophic community members (Table S3). Classification using GTDB (Chaumeil *et al*., 2019) placed the MAG within the genus *Methyloglobulus*. Using the UBCG2 pipeline for bacterial phylogeny our MAG was placed close to other *Methyloglobulus* bins deposited to NCBI's Assembly database (Fig. 4). The two closest assemblies were obtained from samples of activated sludge and a drinking water treatment plant biofilm. Direct average nucleotide and amino acid identities to the *Methyloglobulus morosus* KoM1 reference genome and the obtained MAG resulted in values of 76.8% and 75.5% respectively. Because the genus *Methyloglobulus* currently has one isolated representative, we annotated our MAG to inspect the metabolism of the biofilm methanotroph. No soluble methane monooxygenase was identified, but one *pmoCAB* operon and two *pxmABC* operons were found like in *M*. *morosus* KoM1 (Poehlein *et al*., 2013). Being a type I methanotroph, a ribulose monophosphate pathway for carbon assimilation, respiratory chain and tricarboxylic acid cycle were encoded in the MAG. *Methyloglobulus morosus* KoM1 encodes a nitrogenase for fixing atmospheric nitrogen (Poehlein *et al*., 2013), but none was present in our MAG. A nitrite reductase (*nirB*) was annotated, conferring the ability to respire in the absence of oxygen. No genes for methylphosphonate metabolism were present, unlike the type strain. A sulfide:quinone oxidoreductase was identified as a possible way to circumvent sulfide toxicity. For the second step in methanotrophy, only a lanthanide‐dependent XoxF‐type methanol dehydrogenase was identified, making this another methanotrophic MAG without a calcium‐dependent methanol dehydrogenase (Fig. 5; Keltjens *et al*., 2014; Picone and Op den Camp, 2019). The low coverage of the MAG and the lack of other *pmoA* genes suggest that the canal walls are an ecological niche for methanotrophs and that *Methyloglobulus* is a key community member for CH_4_ metabolism.

## Discussion

The Netherlands is a densely populated river delta, with large parts of the country lying below sea level (Wong *et al*., 2007). During the development of Dutch cities the canals served to optimise land use while allowing for water drainage, thereby preventing flooding of the cities (Hoeksema, 2007). Furthermore, transport of goods using waterways is efficient and access to trade routes was vital for economic development (Klitgaard, 2019). Therefore, man‐made urban canals became ubiquitous in the larger cities and iconic for the Dutch cityscape, and indeed in many cities around the world. At the same time, urban aquatic systems like these canals are implicated to emit CH_4_ (Zazzeri *et al*., 2017; Wang *et al*., 2018; Brigham *et al*., 2019; Herrero Ortega *et al*., 2019; R. Wang *et al*., 2020). Understanding the microbiology behind CH_4_ emissions provides vital information about ecosystem carbon cycling and can aid in designing adequate measures to reduce CH_4_ emissions. We set out to describe the microbial community in the urban canals of Amsterdam, determine the potential for both CH_4_ production and consumption, and to identify an urban niche for CH_4_‐cycling microorganisms.

Urbanisation is linked to eutrophication, with an increasing number of studies reporting increased nutrient load caused by anthropogenic land use (Harrison *et al*., 2012; Gessner *et al*., 2014; Brown and Hershey, 2019; Herrero Ortega *et al*., 2019). Increased nutrient loads can lead to algal blooms in freshwater due to increased net primary production (Huettel *et al*., 2014; Martinez‐Cruz *et al*., 2017; Van Bergen *et al*., 2019). Consequently, the potential for CH_4_ production increases as excess carbon is available, especially in highly eutrophic systems. The data presented here suggest that the Amsterdam canal waters are oligotrophic and oxygenated during summer. Moreover, the lack of CH_4_ production over 100 days from unamended sediments indicates that the top layer canal sediment was depleted of easy‐to‐use carbon. However, the amount of time the sediments were in storage prior to the start of the incubation could have been sufficient to deplete most of the organic matter. The observed CH_4_ production within a week in amended sediment microcosms shows a metabolically active and adaptable methanogenic community. Due to the oxygenated water column and active production of CH_4_ after substrate amendment, the upper layer of the sediment could be an environmental niche for aerobic methanotrophs. Taken together, both the methanogenic and methanotrophic communities are able to respond rapidly to changes in substrate availability and show high potential for being a CH_4_ source and filter respectively.

Methanotrophy in freshwaters has been extensively studied for stratified lakes, while knowledge on riverine systems and shallow lakes is limited (Deutzmann *et al*., 2014b; Oswald *et al*., 2017; Crevecoeur *et al*., 2019; Cabrol *et al*., 2020; Reis *et al*., 2020). The canals of Amsterdam are well‐mixed due to boat traffic, especially in the city centre. Moreover, no floating vegetation was observed which is an important habitat for plant‐associated methanotrophic bacteria in other waters (Kip *et al*., 2011; Faußer *et al*., 2012; Yoshida *et al*., 2014). Instead, we observed that the biofilm alongside the canal wall was capable of rapid oxidation of CH_4_ compared to the water column samples. The brick canal wall is a unique, man‐made structure that is unlike the littoral zone of natural waters and is most commonly found in urban waterways. The rough surface of a clay brick provides opportunity for microorganisms to attach and colonise. Moreover, this brick could be the source of the rare earth elements required for the XoxF‐type methanol dehydrogenase found in the MAG. The canal wall biofilm has the capability of providing niches for diverse microbial metabolisms, niches that might be smaller in more natural settings (Battin *et al*., 2016). In the environmental biofilm sample, 16S rRNA gene sequencing and metagenomics revealed that a *Methyloglobulus* sp. constituted about 0.2%–0.7% of the bacterial community. This low abundance led to low coverage in our metagenome and an incomplete MAG. However, *pmoA* and 16S rRNA phylogeny as well as two separate classification tools placed it within the *Methyloglobulus* genus. Previous studies have found these methanotrophs in lakes (Deutzmann *et al*., 2014a) and sand filters of drinking water treatment plants (Parks *et al*., 2017; Poghosyan *et al*., 2020). Thus, we are the first to report a *Methyloglobulus* sp. in an urban aquatic system and our microcosm experiments showed that these bacteria are active or highly adaptable. We posit that the canal wall biofilms could play an important role in an urban waterway as a niche habitat for CH_4_‐cycling microorganisms.

The initial rates of CH_4_ oxidation in the biofilm were 70 times greater per g_DW_ than the sediment. From the metabolic potentials, the canal wall biofilm seemed to be an environment most suitable for aerobic methanotrophs in our incubation experiments, more so than the sediment or the water column. The biofilm's rates are much higher due to the nature of our drying methods and the normalisation as a biofilm is high in microbial mass, whereas the sediment is higher in non‐microbial mass. The sediment CH_4_ oxidation rates were similar to lakes in Northern Germany (Eller *et al*., 2005). CH_4_ oxidation rates of the sediment were also in line with restored peatland sediment incubations (Reumer *et al*., 2018). However, oxidation potential measured for permafrost wetlands in Siberia exhibited initial rates that were 10 times higher than our sediment incubations (Knoblauch *et al*., 2008). Taken together, aerobic methanotrophic rates in Amsterdam sediments were in the expected range for methanogenic sediments. To our knowledge, this is the first study where a canal wall biofilm has been identified as a habitat with high methanotrophic potential.

Another aspect to the biofilm is its apparent versatility to changes in substrate availability. In theory, many urban surfaces have the potential for biofilm development. Within Amsterdam, this might not be limited to the brick canal wall as there are wooden poles for boat signs, houseboats, concrete walls and steel sheet piles. Consequently, there may be more unique urban habitats where methanotrophs could reside. Methanotrophic biofilms could be a way to mitigate CH_4_ emissions in urban waterways, for example in areas impacted by diffuse pollution from wastewater. However, ebullition could contribute significantly to net CH_4_ emissions in urban waterways as it has been shown to become the dominant emission pathway of methane in natural freshwater ecosystems under warming scenarios (Aben *et al*., 2017). CH_4_ bubbles will not be accessible to the biofilm community in such shallow waters as canals. Indeed, *in situ* measurements indicate that there was excess dissolved CH_4_ (Table 1). Whether due to ebullition or diffusive transport limitations from the water to the canal wall, the biofilm's metabolic capacity was not great enough to mitigate CH_4_ emissions entirely. We conclude that the biofilm community could be a novel CH_4_ filter in urban waters for which stimulation could lead to a greater filter capacity.

We used two different primer sets for archaeal and bacterial 16S rRNA gene amplicon sequencing respectively, to eliminate potential biases and obtain an accurate view of the microbial diversity. In the archaeal domain, the most abundant class was Bathyarchaeia with 31%–41% relative abundance. Due to improvements in sequencing technologies, Bathyarchaeia have been observed in many soils and sediments but their ecological role remains elusive (Zhou *et al*., 2018). These putative organic matter degraders were shown to be able to grow on lignin (Yu *et al*., 2018b). Bathyarchaeia were detected in freshwater lakes and wetlands with similar relative abundances compared to the Amsterdam canals (Yang *et al*., 2016; Narrowe *et al*., 2017). Furthermore, the canal sediment archaeal communities harboured up to 33% CH_4_‐cycling archaea (Fig. 2). The methanogenic community in the canal sediment consisted of a mix of hydrogenotrophic and acetoclastic families. *Methanoregulaceae* were most abundant which is expected due to their ubiquity in freshwater sediments (Wen *et al*., 2017). This family consists of hydrogenotrophic methanogens but was not enriched during our microcosm incubations with H_2_ and CO_2_. Instead, several *Methanobacterium* spp. were enriched, probably favouring the high substrate conditions created in the microcosm incubations. *Methanosaetaceae* were the second most abundant methanogenic family in the Amsterdam canal sediment. They were enriched in microcosms amended with acetate, which is their sole carbon and energy source (Jetten *et al*., 1992; Smith and Ingram‐Smith, 2007). *Methanosaetaceae* have been found in other freshwater sediments like thermokarst lakes and rivers (De Jong *et al*., 2018; Wilkinson *et al*., 2019). The microcosms amended with methanol showed an archaeal community dominated by *Methanosarcinaceae*. Methanogens of this methylotrophic family comprised less than 1% of archaeal sequences in the environmental sediment but were revived quickly in our incubations. Curiously, the community present at the end of the unamended sediment incubations was highly similar to the environmental sediment. This could indicate a carbon‐starved but active methanogenic archaeal community in the sediments because CH_4_ production was observed quickly and their relative abundance did not change over a period of 100 days of incubation in the controls. Importantly, it shows that the incubation strategy employed is relevant to the real‐world situation. Therefore, we hypothesise that acetoclastic and hydrogenotrophic methanogenesis are the dominant CH_4_ production pathways in these urban sediments based on the abundance and activity of the *Methanosaetaceae* family and the presence of *Methanoregulaceae*.

Initial methanogenic rates of the amended sediments were comparable to those of amended Arctic sediments at 20°C (Blake *et al*., 2015). Furthermore, lake sediment from Northern Germany showed similar production rates after acetate amendment (Eller *et al*., 2005). Interestingly, our microcosm incubations had higher initial CH_4_ production than the observed maximum for thermokarst lake sediment (De Jong *et al*., 2018). Thus, our determined methanogenic rates are within the range expected for freshwater sediment after substrate amendment. Unamended sediment incubations did not show CH_4_ production so identifying the source of sediment carbon is a point for further research.

The bacterial community of the environmental sediment was highly diverse, with approximately 40% of the community consisting of sequences with a relative abundance below 1%. Sulfate‐reducing bacteria were abundant, with members of the uncultivated Thermodesulfovibrionia class making up 8%–10% of the total bacterial community (Fig. S4). Sulfate is a byproduct of organic matter degradation and is most likely naturally available in canal sediments (Table S1). The canals of Amsterdam receive brackish water from the IJ, which would increase the sulfate load and, in turn, explain the presence of sulfate reducers. Since the community in the sediment did not change during the microcosm incubations it is likely that the top layer prokaryotic community is probably starved for nutrients. The sediment did not harbour many nitrogen‐cycling microorganisms, with ammonium oxidisers (*Nitrosomonadaceae*) being the most abundant with 1.9%–3.1% relative abundance. Anammox bacteria of the *Brocadiaceae* family comprised less than 0.05% of the total community while no *Nitrobacter* reads were obtained. Nitrite‐oxidising bacteria of the *Nitrospira* genus were detected at 0.8% relative abundance on average, but only in the canal sediment. In summary, nitrogen compounds seem to be present in low amounts indicating that there is little nitrogen pollution even in the Amsterdam city centre.

The genomic potential for anaerobic oxidation of methane was striking. 9% of the archaeal community was classified as *Ca*. Methanoperedens, a methanotroph capable of oxidising CH_4_ anaerobically using NO_3_^−^, Fe(III), or Mn(IV) (Haroon *et al*., 2013; Cai *et al*., 2018; Leu *et al*., 2020). In addition, members of the Methylomirabilota that are known to perform nitrite‐dependent anaerobic methane oxidation were detected to be as much as 1% of the bacterial community (Raghoebarsing *et al*., 2006; Ettwig *et al*., 2010). Linking these two domains of life with the qPCR results (Fig. S8) and metagenome sequencing (Supplementary Tables S4–S6) showed that nitrate‐ and nitrite‐dependent anaerobic methanotrophs occurred at the same approximate absolute abundance. It has been shown that these two anaerobic methanotrophs co‐occur in freshwater sediments and together perform CH_4_‐dependent denitrification (Shen *et al*., 2017; Shen *et al*., 2019). They could fill a niche in the sediment oxidising CH_4_ anaerobically while competing for nitrate with nitrogen‐cycling microorganisms like anaerobic denitrifiers.

Our community analysis and microcosm incubation experiments showed little variation between the sampling sites. The biofilm community was highly similar between the five biological samples and a similar result was observed for the three sediment communities. Even though the environmental samples were taken on opposite sides of the city centre (Fig. S1), their core microbial communities remained comparable. This finding indicates that our studied waterways are spatially homogeneous. Consequently, we propose that our findings are representative for the entire canal network of the Amsterdam city centre. More importantly, our data have the potential to be applicable to other cities with similar canal networks. Cities with eutrophic waterways or agricultural ditches rich in nitrogen and phosphorus will likely have different CH_4_ dynamics from the studied Amsterdam canals. Therefore, investment in efficient wastewater treatment, and the separation of sewer and stormwater systems, could lead to oligotrophic waters and thus lower GHG emissions. However, the exact impact on the microbial community of urban land use compared to other land use types requires further study.

Due to the widespread nature of urban waterways not only in the Netherlands but globally, understanding this ecosystem's response to climate warming and human activity is crucial. Moreover, ecological niches present in urban waterways will likely become more important as more land area will become urbanised. Within this man‐made environment, we found that the biofilm attached to the canal walls has the potential to act as a CH_4_ filter. The activity of the methanogenic community and metabolic potential emphasised that the canals can be a significant source of atmospheric CH_4_. Further research is required to determine if net GHG fluxes and the prokaryotic community changes temporally, especially between summer and winter, and the implications for CH_4_‐cycling and net emissions.

## Methods

### Study site and sampling strategy

The city centre of Amsterdam, the Netherlands, was chosen for sampling (five sites, Fig. S1) due to its large canal network of over 100 km in length. Since the city was founded around 1250 CE, canals have formed an integral part of the urban landscape. Canals are flanked by streets, and boat traffic on the canals is present year‐round. Two main sources of water feed into the canal network; the brackish IJ in the north and the river Amstel in the south. Three types of environmental samples were taken: (i) canal sediment top layer, (ii) canal water and (iii) canal wall biofilm. Sampling was done at each site while on a boat near the canal wall in early July 2019. Water was collected in autoclaved 1 L glass bottles by filling them completely with water about 20 cm under the water–air interface. Filled bottles were closed while submerged and stored on ice. Canal wall biofilm was collected by scraping using an alcohol‐sterilised spatula and transferring it to a sterile 50 ml centrifuge tube. Sediment was collected up to approximately 10 cm depth using a Van Veen grab sampler. Two independent sediment grabs were pooled and transferred immediately to a sterile 50 ml tube (VWR, Amsterdam, Netherlands). All tubes were transported on ice and quickly stored at 4°C until processing.

### Water physicochemical analysis

During sample collection, canal water EC, temperature, depth and dissolved oxygen were measured *in situ* using a KorEXO3 Multiparameter Sonde (YSI, Yellow Springs, OH, USA). Dissolved CH_4_ was measured using headspace extraction from 30 ml surface water as described previously (Dean *et al*., 2020). Briefly, the canal water was sampled using a 60 ml syringe (VWR) and mixed with ambient air by vigorous shaking for 1 min. The headspace was injected under overpressure into a pre‐evacuated Exetainers (Labco, Lampeter, United Kingdom). After transporting the samples to Radboud University (Nijmegen, Netherlands), the absolute CH_4_ in the Exetainers was measured on a gas chromatograph equipped with a Porapak Q‐column (100/120 mesh) and a flame ionisation detector (HP 5890 series II; Agilent Technologies, Santa Clara, CA, USA) by triplicate injections of 50 μl. CH_4_ was calculated based on a calibration curve from 0.03 to 10 mmol L^−1^ CH_4_ in a headspace. TOC and total nitrogen were determined using a TOC‐L CPH/CPN analyser (Shimadzu's‐Hertogenbosch, Netherlands) for the canal water samples. NO_3_‐N, NH_4_‐N, PO_4_‐P and Cl^−^ were measured using colorimetric assays on an AutoAnalyzer3 (Bran+Luebbe, Norderstedt, Germany). Na and K were measured using a flame‐photometer (Sherwood Scientific, Cambridge, United Kingdom). 10 ml canal water samples were acidified to 1% nitric acid and analysed for Al, As, B, Ca, Cd, Co, Cr, Cu, Fe, Hg, K, Mg, Mn, Mo, Na, Ni, P, Pb, S, Si, Sr and Zn using inductively coupled plasma‐optical emission spectrometry on an iCAP 6000 (Thermo Fischer Scientific, Bremen, Germany).

### Methanogenic incubations

Sediments were kept at 4°C after sample collection and incubations were started within 1 month. The sediments were slurried 1:6 wt./vol. with unsterilized canal water from the respective sample site. Unsterilized canal water was used as it was not expected to interfere with the anaerobic incubations as we hypothesised that the aerobic water column would not be a source of anaerobic microorganisms. Slurries were sieved through a clean mesh with a 1.5 mm pore size to remove debris that would not fit through the opening of the 120 ml serum bottles while retaining the majority of the soil particles. 1 ml aliquots were taken to determine the dry weight of the slurry by drying at 70°C for 3 days. 20 ml slurry was added to sterilised 120 ml serum bottles and closed with red butyl rubber stoppers that were boiled three times in water. Aluminium crimp caps were used to keep the stoppers in place. Anoxic conditions were achieved by creating a vacuum in the headspace and gassing with argon using a 0.5 bar overpressure. Four vacuum‐gassing cycles of 10 min were deemed sufficient for anoxic conditions. For methanogenic potential on substrates autoclaved stock solution was added using sterile needles and syringes to get 2 mM acetate, 2 mM methanol, and H_2_ (8 mM) and CO_2_ (2 mM) in the liquid phase. CH_4_ production was monitored using a gas chromatograph (HP 5890 series II; Agilent Technologies) by triplicate injections of 50 μl headspace per microcosm. If the amount of CH_4_ was constant over multiple days of measuring, a new substrate was added to the respective incubations. All incubation conditions were performed in triplicate and bottles were measured daily whenever possible. Bottles were placed on a shaking plate at 90 rpm and room temperature (21°C) and shielded from direct light sources with aluminium foil and cardboard.

### Methanotrophic incubations

To assess the maximum methanotrophic potential of the Amsterdam canal samples, we performed aerobic incubations with CH_4_ amendments. Incubations were started within 1 month of field collection. All incubations used sterilised 120 ml serum bottles with a total liquid volume of 20 ml. Environmental biofilm samples were homogenised by hand using a glass tissue grinder (DWK Life Sciences, Mainz, Germany). 1 ml of hand‐homogenised biofilm was added to 19 ml filter‐sterilised canal water from the respective site to prevent cross‐contamination water‐borne bacteria. 1 ml aliquots were taken to determine the dry weight of the homogenised biofilm by drying at 70°C for 3 days. For the canal water incubations, 20 ml of sampled canal water was used per bottle. Per site, 20 ml autoclaved canal water was used as an abiotic control. Initial measurements of CH_4_ consumption were measured by adding 1 mmol CH_4_ to the microcosm headspace. However, this amount of CH_4_ led to oxygen limitation, so subsequent additions of CH_4_ were done after flushing with at least two headspace volumes using filter‐sterilised air. 10 ml CH_4_ and 15 ml ambient lab air were added to keep the initial overpressure at 0.25 bar. Sediment aerobic methanotrophic potential was determined with a 1:15 wt./vol. slurry of canal sediment and filter‐sterilised canal water. Slurries were sieved to remove large debris as described for the methanogenic incubations above. 1 ml aliquots were taken to determine the dry weight of the slurry by drying at 70°C for 3 days. Aerobic sediment incubations were started 2.5 months after field collection. 10 ml pure CH_4_ was added together with 15 ml of ambient lab air for an initial overpressure of 0.25 bar. If no significant residual amount of CH_4_ was measured, the headspace was flushed with at least two headspace volumes of filter‐sterilised air before adding new CH_4_. Every condition per sample site was performed in triplicate. All bottles were incubated on a shaking plate at 90 rpm and room temperature, shielded from direct light sources.

### DNA isolation and 16S rRNA gene amplicon sequencing

Microbial community profiling was done by sequencing 16S rRNA gene amplicons using a DNA template from each biological sample. Water samples were filtered within a day of sampling over 0.22 μm Nuclepore track‐etch membrane filters (Whatman, Maidstone, United Kingdom). Between 50 and 100 ml of canal water was used depending on how fast the filter blocked. Filters were stored at −20°C until further processing. Molecular analyses of the biofilm samples were done on the hand‐homogenised samples mentioned above. Around 0.5 g of sediment was used to isolate DNA for the canal sediment samples. All samples were processed within 15 days of sampling. To determine the effect of the microcosm incubations on the community composition, DNA was extracted from the microcosms after three substrate additions. For sediment microcosms, 300 μl of slurry was used. The suspended solids in each biofilm microcosm were decanted into a centrifuge tube and centrifuged at 4000 rpm for 1 min. The supernatant was decanted and the cells freeze‐dried for storage and dry weight determination. DNA was isolated for all samples using the DNeasy PowerSoil DNA Isolation kit according to the manufacturer's instructions (Qiagen, Venlo, Netherlands), with the alteration that the PowerBead tubes were bead‐beated on a TissueLyser LT (Qiagen) for 10 min at 50 Hz and the DNA was eluted using two elution steps with 25 μl autoclaved ultrapure water. Eluted DNA was stored at −20°C until sequencing. 16S rRNA gene amplicon sequencing was done by Macrogen (Macrogen, Amsterdam, Netherlands) using the Illumina MiSeq Next Generation Sequencing platform. Paired‐end libraries were constructed using the Illumina Herculase II Fusion DNA Polymerase Nextera XT Index Kit V2 (Illumina, Eindhoven, Netherlands). Primers used for bacterial amplification were Bac341F (5′‐CCTACGGGNGGCWGCAG‐3′; Herlemann *et al*., 2011) and Bac806R (5′‐GGACTACHVGGGTWTCTAAT‐3′; Caporaso *et al*., 2012). Archaeal amplification was performed with primers Arch349F (5′‐GYGCASCAGKCGMGAAW‐3′) and Arch806R (5′‐GGACTACVSGGGTATCTAAT‐3′; Takai and Horikoshi, 2000). The obtained raw reads have been deposited in the European Nucleotide Archive under the accession number PRJEB40426 (https://www.ebi.ac.uk/ena/browser/view/PRJEB40426).

### Amplicon sequencing data analysis

Raw sequencing reads were checked for quality using FastQC (v0.11.5; Andrews *et al*., 2010). The bacterial dataset showed contamination of transposase adapters which was removed using Cutadapt (v1.18; Martin, 2011). Approximately 95 000 Between 82 000 and 110 000 paired‐end bacterial or archaeal sequencing reads were obtained per sample. Data were further processed using the DADA2 pipeline (v1.8; Callahan *et al*., 2016) in R (v3.5.1; R Core Team, 2019). Species taxonomy was assigned using the SILVA 16S rRNA database release 138.1 (Quast *et al*., 2012). Microbial community data were analysed and visualised using the R package phyloseq (v1.30.0; McMurdie and Holmes, 2013). Absolute abundance of bacteria and archaea was measured with qPCR using the same primer pairs used for 16S rRNA gene amplicon sequencing as they were shown to have little cross‐reactivity (Klindworth *et al*., 2013). A single copy of a 16S rRNA gene was cloned into a pGEM‐T Easy vector (Promega, the Netherlands) and used to produce a standard curve. DNA concentrations were measured with the Qubit HS dsDNA assay (Invitrogen, USA). qPCR reactions were performed in a C1000 Touch thermocycler with a CFX96 Touch Real‐Time PCR detection system (Bio‐Rad Laboratories, the Netherlands). The reaction mix (25 μl) consisted of 12.5 μl PerfeCTa Quanta SYBR Green FastMix (Quanta Bio, USA), 0.4 pmol μL^−1^ of both the reverse and forward primers, 1 μl of template DNA and sterile ultrapure water. The following programme was used: initial denaturing of the DNA for 3 min at 95°C; 40 cycles of 30 s at 95°C, 30 s at 58°C and 30 s at 72°C with a plate read; after 40 cycles a melt curve from 30 to 95°C with increments of 0.5°C was measured to check for PCR specificity. For the archaeal 16S rRNA gene qPCR reactions, the annealing temperature was adjusted to 60°C. Each plate was run with a duplicate standard curve ranging from 10^2^ to 10^9^ copies of the 16S rRNA gene. The slope of the standard curve was used to calculate the PCR efficiency and plates were considered unreliable if this number was lower than 90%.

### Whole metagenome sequencing, genome binning and sequence analysis

DNA from the environmental sediment and biofilm, extracted as outlined above, was used for full metagenome sequencing to get a broader view of the microbial communities in Amsterdam's canals. Sequencing was performed by Macrogen (Macrogen) using the Illumina NovaSeq 6000 platform and the TruSeq DNA Nano library preparation kit, yielding 150 bp paired‐end reads and in total about 16 400 000 sequencing reads per sample. An automated pipeline for binning was used to co‐assemble the three sediment samples and five biofilm samples. In short, trimming and quality filtering is performed by BBDuk (BBTools v38.75). Error correction was applied to the trimmed and filtered reads using BayesHammer (Nikolenko *et al*., 2013). *De novo* co‐assembly was done using MEGAHIT (v1.2.9; Li *et al*., 2015) using k‐mer sizes 21,29, 39, 59, 79, 99 and 119. Read mapping onto the assembled metagenomes was handled by BBMap (BBTools v38.75). Binning was done by aggregating results from BinSanity (v0.3.1; Graham *et al*., 2017), CONCOCT (v1.1.0; Alneberg *et al*., 2014), MaxBin2 (v2.2.7; Wu *et al*., 2015) and MetaBAT2 (v2.15; Kang *et al*., 2019) using DAS Tool (v1.1.2; Sieber *et al*., 2018) generating consensus bins. Finally, the quality of the consensus bins was determined using CheckM (v1.1.2; Parks *et al*., 2015). Taxonomy of the consensus bins was cross‐validated using the Genome Taxonomy Database Toolkit (v1.3.0; Chaumeil *et al*., 2019). Raw metagenome reads were deposited to the European Nucleotide Archive under the project number PRJEB40426 (https://www.ebi.ac.uk/ena/browser/view/PRJEB40426).

16S rRNA gene sequences were extracted from the raw reads using phyloFlash (v3.4; Gruber‐Vodicka *et al*., 2020). In addition to metagenome binning, coding genes were identified from the raw data using Prodigal (v2.6.3; Hyatt *et al*., 2010). Subsequently, HMMER (v3.3; Eddy, 1998) was used to identify *pmoA*, *mdh* and *mcrA* sequences using profiles from the Pfam database (El‐Gebali *et al*., 2018). The *pmoCAB* operon was identified from the metagenome reads after read mapping to the *Methyloglobulus morosus* KoM1 reference genome and assembling with SPAdes (v3.14.0; Prjibelski *et al*., 2020). Prokka (v1.14.6; Seemann, 2014) was used to annotate the obtained bin using the BLASTp RefSeq database (O'Leary *et al*., 2015). Using the Prodigal predicted amino acid output, the average amino acid identity to the *M*. *morosus* KoM1 reference genome was computed using CompareM (https://github.com/dparks1134/CompareM). A phylogenetic tree of the obtained methanotroph MAG was constructed using UBCG2 (Kim *et al*., 2021) and 578 GenBank assemblies downloaded from NCBI (https://www.ncbi.nlm.nih.gov/assembly). UBCG2 calculates an index based upon the amount of ‘core genes’ that support the tree branch structure. This index is the gene support index and has a maximum value of 81. The phylogenetic tree was visualised using the R package ‘ggtree’ (Yu, 2020). The MAG can be accessed from the European Nucleotide Archive under project number PRJEB40426.

## Supporting information


**Appendix** S1: Supporting information.
