# Supplementary material for: Amsterdam urban canals contain novel niches for methane‐cycling microorganisms
Source: Environ Microbiol. 2021 Dec 13;24(1):82–97. doi: 10.1111/1462-2920.15864 (PMC9299808; doi:10.1111/1462-2920.15864)
Supplement: Supplementary file 1 — Appendix S1: Supporting information. [file EMI-24-82-s001.pdf]

Supplementary Methods

To calculate the amount of CH_4 inside our microcosm incubations we combine the amount of headspace CH_4 and the dissolved CH_4 using Henry's law (Equation 1). We calculate the absolute amount of CH_4 in mmol using our calibration curve on the average of three triplicate injections of each microcosm. Additionally, we corrected for the headspace pressure by multiplying the volume with the total pressure inside our bottle (Equation 2), measured after each measuring point with a digital pressure meter (GMH 3111, GHM Messtechnik GmbH, Regenstauf, Germany). We calculated the partial pressure of CH_4 inside each microcosm with the ideal gas constant (R), a temperature of $20\,°C$ (T, or $293.15\,K$) and the total pressure (Equation 3). The partial pressure was, subsequently, calculated using the law of partial volumes (Equation 4). Finally, the total amount of CH_4 in the microcosm is the addition of the headspace CH_4 and the dissolved CH_4 (Equation 5). All incubation data shown is the average and standard deviation of the mean of triplicate incubations.

$$[CH4]_{liq} = p_{CH_4} K_H \tag{1}$$

$$CH_{4,abs,HS} = \frac{Peak\,area}{Calibration\,curve} V_{HS} P_{tot} \tag{2}$$

$$V_{CH_4} = \frac{CH_{4abs,HS} RT}{P_{tot}} \tag{3}$$

$$p_{CH_4} = \frac{(V_{CH_4} \times 10^{-3}) P_{tot}}{V_{HS}} \tag{4}$$

$$CH_{4,tot} = CH_{4abs,HS} + ([CH4]_{liq} V_L) \tag{5}$$

Supplementary Figures

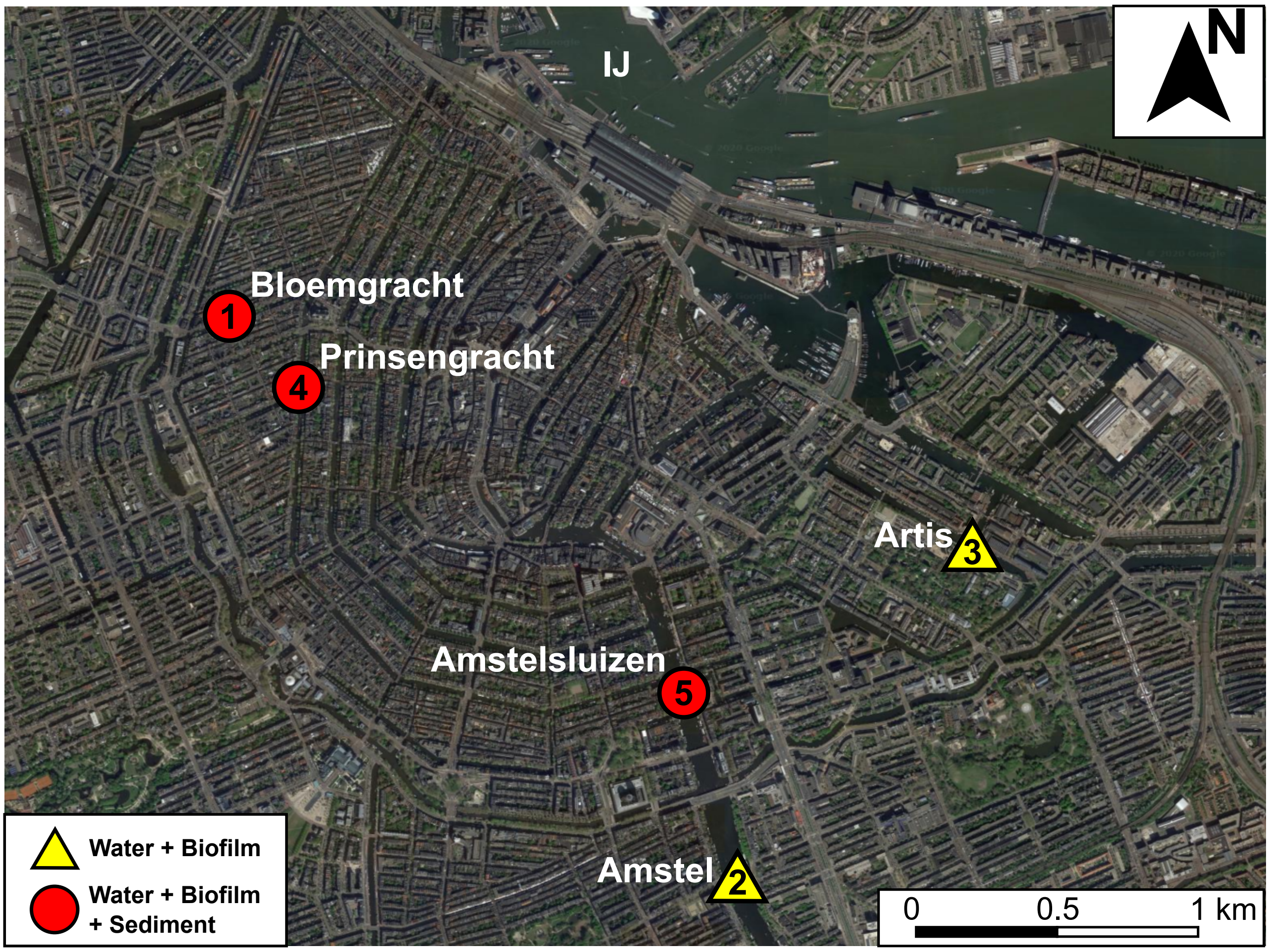

Figure S1. Geographical overview of sampling locations within the city centre of Amsterdam, the Netherlands. Satellite image acquired from Google Earth.

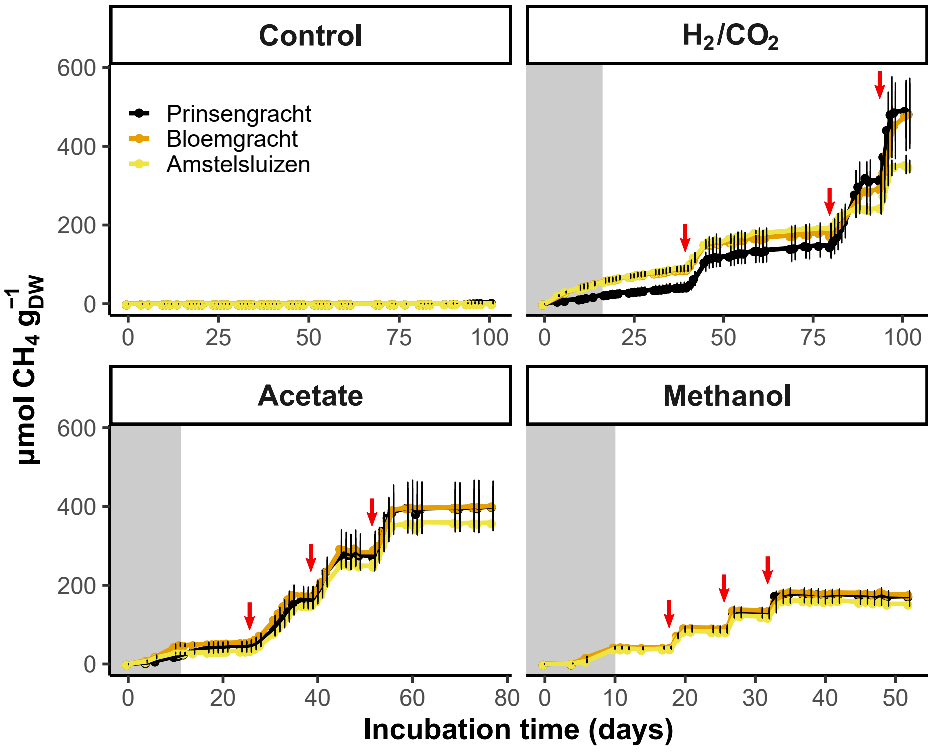

Figure S2. Production of CH_4 over time for the methanogenic incubations of sediment from three different Amsterdam city centre canals. All incubations were performed at room temperature. Each data point represents the average of triplicates with the corresponding standard deviation shown as error bars. Sediment samples were amended with three different substrates: a mixture of hydrogen and carbon dioxide (8 mM H_2/ 2 mM CO_2), methanol (2 mM MeOH) and acetate (2 mM). Endogenic CH_4 production was measured in unamended control incubations. Substrate additions are indicated by the red arrows. The timeframe used for the calculation of the initial production rate is shaded in grey.

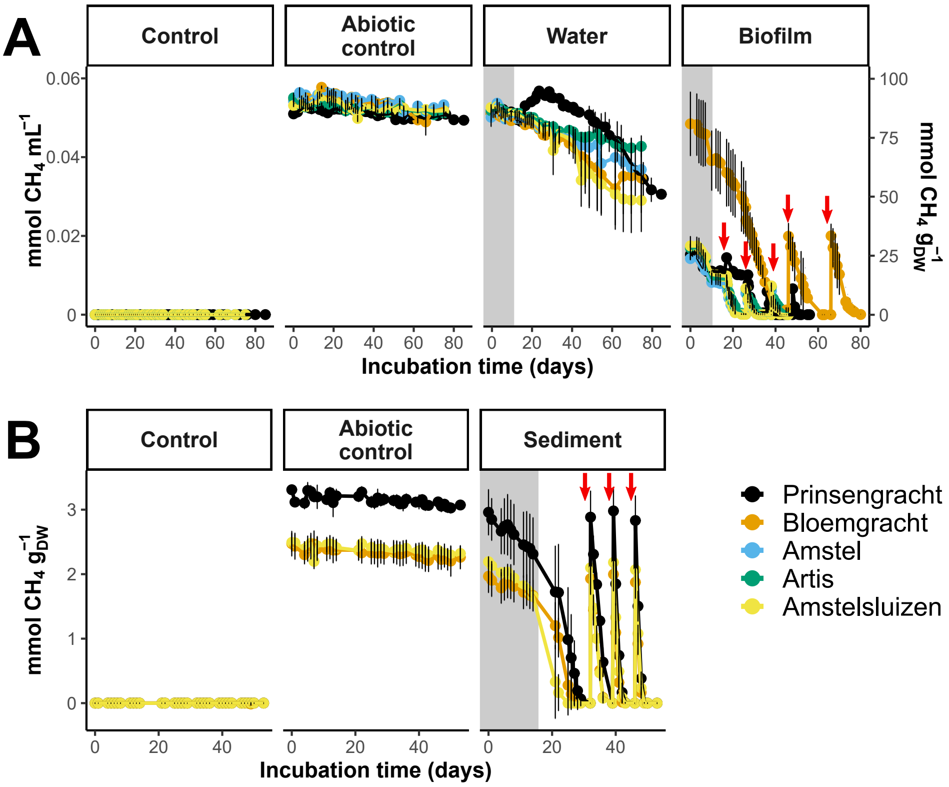

Figure S3. Consumption of CH_4 in microcosm incubations of Amsterdam canal wall biofilm (**A**), and water and canal sediment (**B**). An abiotic control was taken along by autoclaving the respective sample dilutions and adding CH_4 to the headspace. Once CH_4 consumption stopped, the headspace was flushed with sterile air and more CH_4 was added to continue the incubation. Each data point represents the average of triplicates with the corresponding standard deviation shown as error bars. Substrate additions are indicated by the red arrows. The timeframe used for the calculation of the initial oxidation rate is shaded in grey.

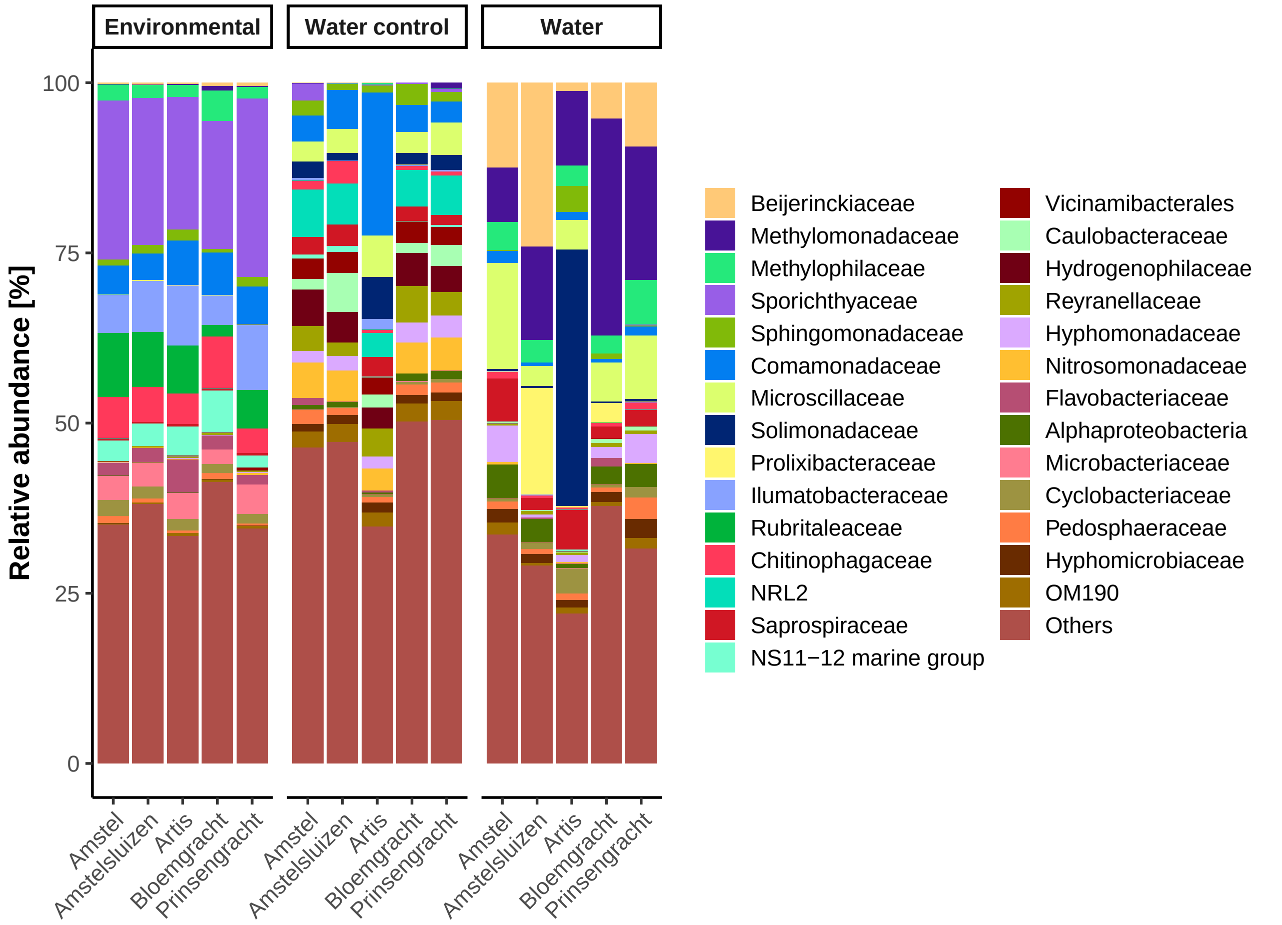

Figure S4. Bacterial 16S rRNA gene ASVs obtained for the incubations of the canal water. Maximum classification depth is on family level. ASVs present in less than 1% relative abundance are grouped in "Others".

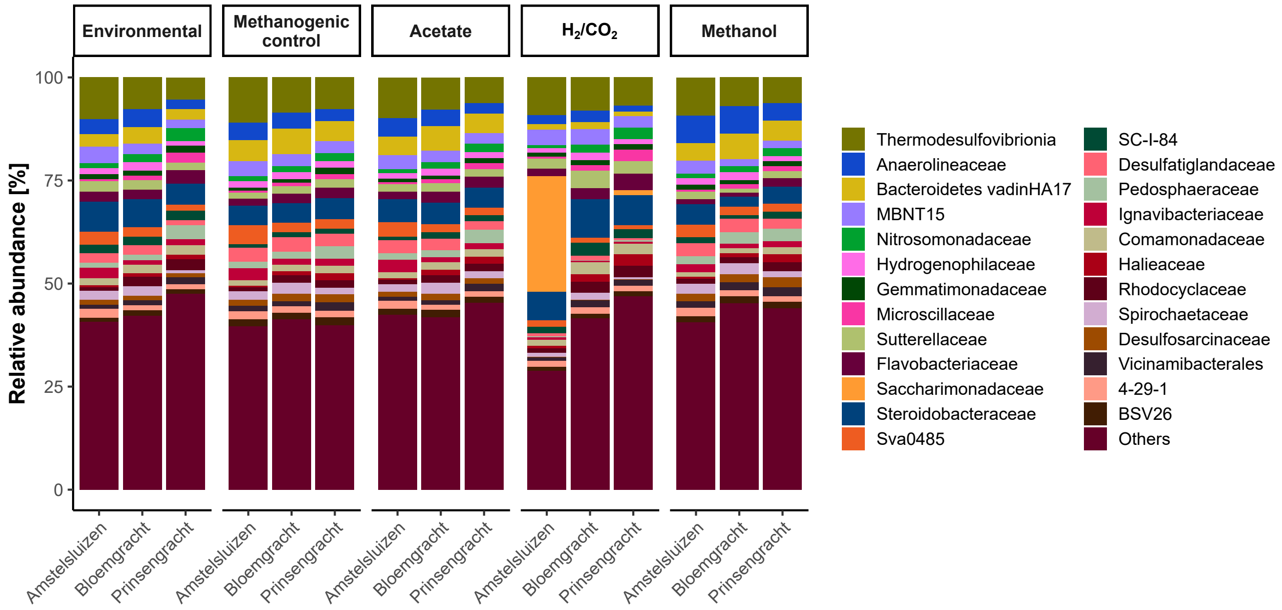

Figure S5. Bacterial 16S rRNA gene ASVs obtained for the methanogenic incubations of the canal sediment. Maximum classification depth is on family level. ASVs present in less than 1% relative abundance are grouped in "Others".

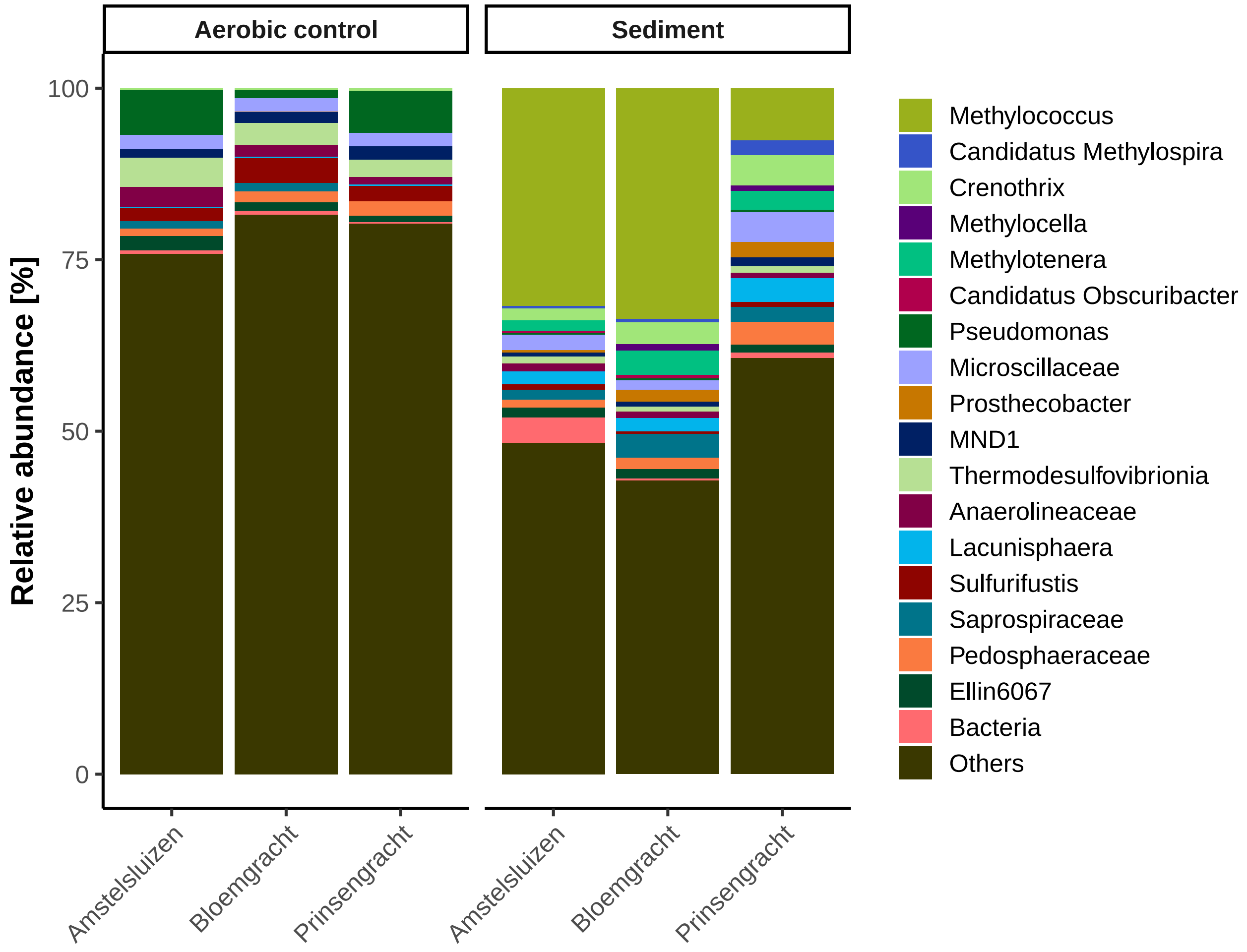

Figure S6. Bacterial 16S rRNA gene ASVs obtained for the methanotrophic incubations of the canal sediment. Maximum classification depth is on family level. ASVs present in less than 1% relative abundance are grouped in "Others".

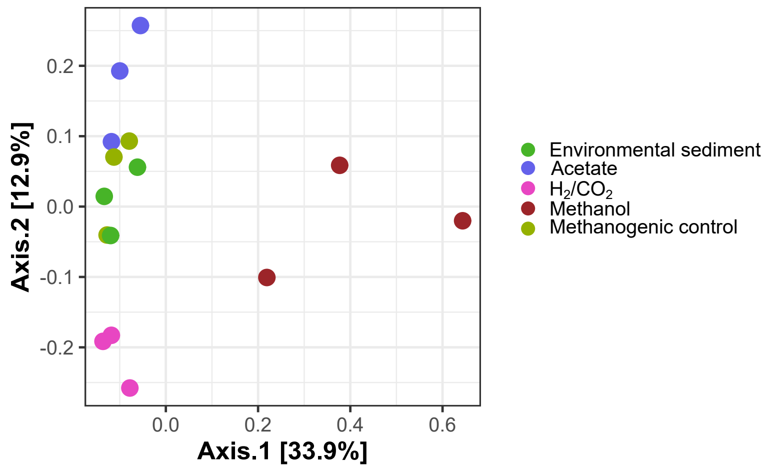

Figure S7. Principal coordinate analysis of all archaeal ASVs for all samples and incubations. Ordination was performed based on Bray-Curtis dissimilarity. Colours represent the different amendment conditions.

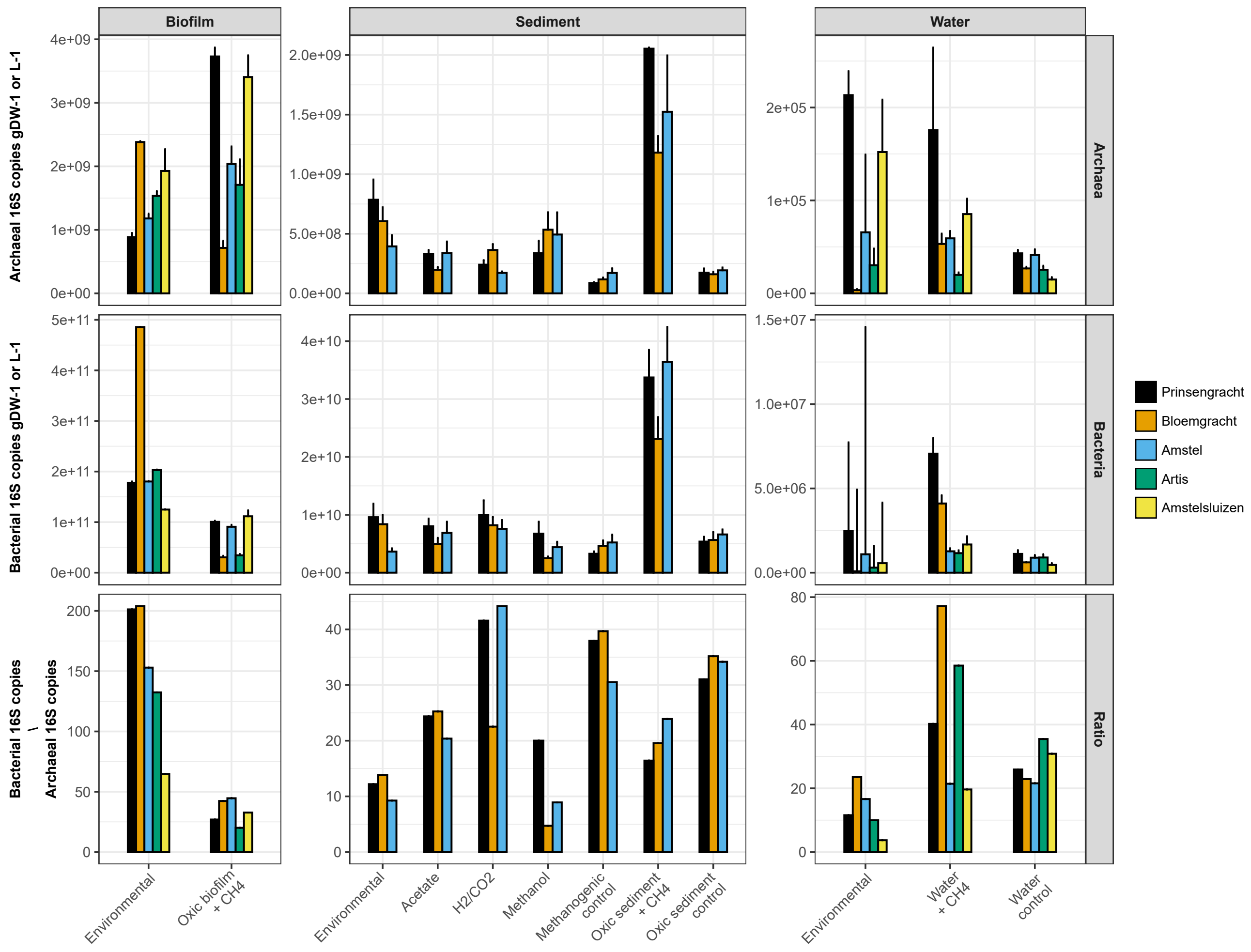

Figure S8. Absolute abundances of bacteria and archaea as determined by qPCR amplifying the 16S rRNA gene. The ratio was calculated as bacterial copies divided by archaeal copies. Therefore, a ratio greater than 1 means more bacterial 16S copies than archaeal 16S copies. Copies of the water column are expressed in L^{-1} and sediment or biofilm copies as ${g_{DW}}^{-1}$. Details on the qPCR conditions are presented in the Methods section of the main text.

Supplementary Tables

Table S1. Metal and elemental composition of the water column as measured by ICP-OES. All values are in ppb.

Site	Al	As	B	Ca	Cd	Co	Cr	Cu	Fe	Hg	K	Mg	Mn	Mo	Na	Ni	P	Pb	S	Si	Sr	Zn
Bloemgracht	55.4	0.0	150.0	84890	0.1	0.9	0.8	5.7	111.0	0.1	10880	31850	285.7	1.1	207400	2.5	158.8	0.0	27060	533.1	483.9	14.8
Prinsengracht	43.5	3.3	143.4	81270	0.2	0.2	0.1	5.6	108.7	0.0	10690	33400	307.7	1.2	214300	3.1	152.7	0.0	29150	394.8	477.6	16.7
Artis	43.9	5.3	254.3	79960	0.9	2.2	0.0	11.3	123.1	0.0	20020	79730	232.3	3.0	466000	4.5	107.6	4.7	57790	946.0	650.9	16.7
Amstel	96.0	6.6	87.9	70470	0.2	1.8	0.0	1.0	109.9	0.0	6935	18880	172.8	0.0	109300	2.1	118.3	0.0	21270	753.1	395.6	6.0
Amstelsluizen	48.3	3.1	142.5	75580	0.4	1.9	1.7	6.4	172.4	0.0	11170	35490	248.4	1.2	224400	4.3	135.2	6.7	32360	509.0	481.2	9.5

Table S2. General metagenome-assembled genome of the *Methyloglobulus* bin obtained by co-assembling five biofilm metagenomes. Statistics are provided per the MIMAG standard. Coverage is tabulated per metagenome sample.

**MAG MCAMS1**	
Taxonomy (GTDB-Tk)	d__Bacteria;p__Proteobacteria;c__Gammaproteobacteria; o__Methylococcales;f__Methylomonadaceae;g__Methyloglobulus
MAG size [Mbp]	2.52
Scaffolds	691
N50 [bp]	4377
GC content [%]	46.07
Completeness [%]	84.26
Contamination [%]	6.61
5S rRNA	MCAMS1_01688
16S rRNA	Not assembled
23S rRNA	Not assembled
tRNA count	23
Assembly quality	Medium
**MAG coverage**	
Amstel	0.96x
Artis	2.11x
Bloemgracht	0.52x
Amstelsluizen	1.28x
Prinsengracht	0.58x

Table S3. HMMER (v3.3) of profiles targeting *mcrA* (PF02249), *mdh* (PF02315) and *pmoA* (PF14100) from the Pfam database. All genes found were subjected to BLASTp (ncbi.nlm.nih.gov/blast) and the top hit is presented together with query cover, percent identity and accession number.

Gene top hit hit	Taxonomy of top hit	Query cover	Percent identity	Accession number
	mcrA			
Coenzyme-B sulfoethylthiotransferase subunit alpha	Methanomicrobiales archaeon	99%	93.55%	NYT08237.1
Coenzyme-B sulfoethylthiotransferase subunit alpha	Methanosarcinales archaeon	100%	94.20%	TRZ89706.1
Methyl-coenzyme M reductase subunit alpha	*Methanosaeta* sp. PtaU1.Bin112	100%	96.01%	OPY55863.1
	mdh			
	Methyloglobulus sp.	98%	92.47%	NOU22217.1
	pmoA			
Methane monooxygenase/ammonia monooxygenase subunit A	*Nitrospira* sp.	100%	100.00%	HBR51999.1
Methane monooxygenase/ammonia monooxygenase subunit A	*Nitrosomonas* sp. APG5	99%	98.91%	WP_106708387.1
Methane monooxygenase/ammonia monooxygenase subunit A	*Nitrospira* sp.	99%	99.64%	NJL18594.1
Methane monooxygenase/ammonia monooxygenase subunit A	*Nitrospira* sp.	99%	98.21%	MBX3347353.1
Particulate methane monooxygenase subunit beta	*Methyloglobulus morosus*	99%	96.36%	WP_023496393.1
Ammonia monooxygenase subunit	*Nitrosomonas* sp. Nm33	99%	90.49%	SDZ11758.1
Methane monooxygenase/ammonia monooxygenase subunit A	*Nitrosomonas* sp.	99%	97.45%	MBX3639921.1
Methane monooxygenase/ammonia monooxygenase subunit A	*Nitrosomonas* sp. APG5	100%	99.12%	WP_106708387.1
Methane monooxygenase/ammonia monooxygenase subunit A	*Nitrosomonas* sp.	99%	100.00%	MBK9663668.1
Methane monooxygenase/ammonia monooxygenase subunit A	*Methylobacter tundripaludum*	95%	71.73%	WP_006890226.1

Table S3. Comparison of bacterial taxa relative abundances between the metagenome through phyloFlash (v3.3) and the 16S rRNA gene amplicon sequencing of the Amsterdam canal sediment. Proportions of the 16S amplicon data do not sum to 100% due to differences in taxa classification by the different pipelines. n.c.; not classified.

	**phyloFlash**			**16S**		
	Amstelsluizen	Bloemgracht	Prinsengracht	Amstelsluizen	Bloemgracht	Prinsengracht
Bacteria	5.30	4.71	3.92	n.c.	n.c.	n.c.
Bacteroidetes vadinHA17	1.42	1.74	1.28	2.97	4.01	2.52
Flavobacteriaceae	1.03	0.89	1.68	2.42	2.34	3.31
Anaerolineaceae	3.95	4.00	3.41	3.72	4.42	2.29
Desulfatiglandaceae	1.74	1.57	0.88	2.36	2.26	1.25
Gemmatimonadaceae	1.68	1.38	1.68	1.19	1.24	1.66
MBNT15	1.90	1.06	0.81	4.06	2.51	2.08
Thermodesulfovibrionia	5.23	4.03	2.52	10.09	7.68	5.44
Gammaproteobacteria	1.59	1.37	1.74	n.c.	n.c.	n.c.
Burkholderiales	1.21	2.05	2.01	n.c.	n.c.	n.c.
Comamonadaceae	1.29	1.73	2.25	1.66	2.07	2.40
Nitrosomonadaceae	1.21	1.76	2.68	1.23	1.97	3.10
Rhodocyclaceae	0.96	1.88	2.80	0.97	2.18	2.72
SC-I-84	2.48	2.42	2.77	2.01	2.16	2.39
Sutterellaceae	2.33	1.81	1.62	2.50	2.27	1.85
Steroidobacteraceae	6.20	5.64	4.17	7.33	6.87	4.99
Spirochaetaceae	2.18	2.33	0.82	2.21	2.42	0.82
Omnitrophaceae	1.65	1.38	0.59	n.c.	n.c.	n.c.
Pedosphaeraceae	1.32	1.62	2.52	1.13	1.35	3.31
Others	55.33	56.65	59.86	40.68	42.19	47.52

Table S4. Comparison of archaeal taxa relative abundances between the metagenome through phyloFlash (v3.3) and the 16S rRNA gene amplicon sequencing of the Amsterdam canal sediment. Proportions of the 16S amplicon data do not sum to 100% due to differences in taxa classification by the different pipelines. n.c.; not classified.

	**phyloFlash**			**16S**		
	Amstelsluizen	Bloemgracht	Prinsengracht	Amstelsluizen	Bloemgracht	Prinsengracht
Archaea	6.02	1.96	3.40	2.13	2.11	0.93
Altiarchaeaceae	1.53	1.51	0.85	n.c.	n.c.	n.c.
Bathyarchaeia	18.65	18.25	23.83	34.70	37.13	44.45
Methanofastidiosales	1.65	1.96	n.c.	n.c.	n.c.	n.c.
Methanoregulaceae	10.74	11.16	6.81	14.50	14.18	10.68
Methanoperedenaceae	3.90	4.68	3.40	8.56	10.01	9.83
Methanosaetaceae	3.07	4.07	3.40	5.29	6.64	6.09
Woesearchaeales	18.42	20.21	29.36	3.96	3.17	6.04
Marine Benthic Group D and DHVEG-1	5.08	3.32	3.83	9.30	6.42	6.52
Methanomassiliicoccales	7.08	9.80	5.53	n.c.	n.c.	n.c.
Thermoplasmata	1.89	1.36	1.70	3.56	2.37	2.65
Others	21.96	21.72	17.87	5.95	6.29	4.92

Table S5. Comparison of bacterial taxa relative abundances between the metagenome through phyloFlash (v3.3) and the 16S rRNA gene amplicon sequencing of the Amsterdam canal wall biofilm. Proportions of the 16S amplicon data do not sum to 100% due to differences in taxa classification by the different pipelines. n.c.; not classified.

	**phyloFlash**					**16S amplicon**				
	Amstel	Amstelsluizen	Artis	Bloemgracht	Prinsengracht	Amstel	Amstelsluizen	Artis	Bloemgracht	Prinsengracht
Bacteria	3.42	2.99	3.24	2.84	2.47	n.c.	n.c.	n.c.	n.c.	n.c.
Burkholderiales	0.59	1.03	0.70	0.54	0.84	n.c.	n.c.	n.c.	n.c.	n.c.
Chloroplast	2.11	4.23	2.43	1.10	1.77	n.c.	n.c.	n.c.	n.c.	n.c.
Enterobacterales	0.75	0.65	0.94	1.28	0.88	n.c.	n.c.	n.c.	n.c.	n.c.
Gammaproteobacteria	1.21	1.26	1.01	1.17	1.62	n.c.	n.c.	n.c.	n.c.	n.c.
Aeromonadaceae	11.49	3.56	8.15	11.78	5.47	6.45	2.82	5.16	9.61	3.78
Alteromonadaceae	1.20	2.44	2.23	5.08	2.49	n.c.	n.c.	n.c.	n.c.	n.c.
Arcobacteraceae	9.67	1.50	7.31	n.c.	2.57	24.48	3.60	15.61	0.02	5.67
Chitinibacteraceae	1.15	0.36	0.60	0.39	1.82	n.c.	n.c.	n.c.	n.c.	n.c.
Clostridiaceae	2.50	1.93	3.21	0.00	2.80	5.56	3.79	8.15	n.c.	5.47
Comamonadaceae	2.23	6.04	4.74	3.41	7.42	1.50	5.36	3.93	2.73	6.59
Flavobacteriaceae	3.50	13.14	9.05	23.71	13.82	7.20	25.11	15.96	38.89	25.66
Methylomonadaceae	0.47	0.60	0.48	0.54	0.63	0.17	0.73	0.46	0.37	0.47
Moraxellaceae	1.61	0.78	2.81	1.16	10.07	1.61	1.10	3.61	1.41	13.66
Nitrospiraceae	0.94	1.34	0.66	0.75	0.88	n.c.	n.c.	n.c.	n.c.	n.c.
Oxalobacteraceae	1.17	1.75	0.62	2.16	2.57	n.c.	n.c.	n.c.	n.c.	n.c.
Pseudomonadaceae	22.23	9.25	11.21	14.79	11.43	21.84	11.01	11.46	16.33	11.54
Rhodobacteraceae	0.36	1.08	0.99	0.65	0.40	n.c.	n.c.	n.c.	n.c.	n.c.
Rhodocyclaceae	0.55	1.32	1.90	0.27	1.77	n.c.	n.c.	n.c.	n.c.	n.c.
Shewanellaceae	14.07	11.23	16.71	13.82	9.48	14.05	14.95	17.14	16.34	9.18
Steroidobacteraceae	0.61	1.06	0.45	0.35	0.63	n.c.	n.c.	n.c.	n.c.	n.c.
Uncultured bacterium	0.78	1.74	0.97	0.60	0.88	n.c.	n.c.	n.c.	n.c.	n.c.
Xanthomonadaceae	0.43	1.67	0.85	0.68	1.09	0.28	2.03	0.67	0.65	0.90
Others	16.94	29.06	18.73	12.95	16.17	15.32	25.46	15.81	12.32	15.80
